# Biphasic responses of the brachial artery diameter following forearm occlusion: a blunted response in the elderly

**DOI:** 10.1186/1476-5918-5-4

**Published:** 2006-04-05

**Authors:** Devon A Dobrosielski, Arturo A Arce, Jason D Allen, Robert H Wood, Michael A Welsch, the Louisiana Healthy Aging Study

**Affiliations:** 1 Department of Kinesiology, Louisiana State University, Baton Rouge, LA, USA; 2 Division of Cardiovascular Medicine, Department of Medicine, Duke University Medical Center, Durham, NC, USA

## Abstract

**Background:**

The purpose was to examine the temporal response of the brachial artery diameter following 5 minutes of forearm occlusion in young men. A secondary objective was to compare the main features of the temporal pattern between young and old.

**Methods:**

Sixteen young (28 ± 8 yrs) and fifteen older (85 ± 8 yrs) men underwent high-resolution ultrasonography of the brachial artery before and after five minutes of forearm occlusion.

**Results:**

Following release of the pressure cuff the brachial artery diameter exhibits a temporal biphasic response. Initially, there is a significant reduction in brachial diameter (NIL) compared to baseline (BASE), followed by a rapid increase to a PEAK at 41 sec post release. When comparing the magnitude of the decrease in diameter and the Brachial Artery Flow Mediated Dilation (BAFMD) between Young and Old, older subjects demonstrated a blunted response (Magnitude of Decrease: Young: 2.0%; Old: 0.4%, *p *= 0.015, and BAFMD: Young: 7.7%; Old: 2.3%, *p *= 0.001). Finally, a significant relationship was noted between the magnitude of decrease and BAFMD (r = -0.44, *p *= 0.04).

**Conclusion:**

Examination of the temporal response of the brachial artery diameter following 5 minutes of forearm occlusion reveals a biphasic pattern in all participants. Specific features of this pattern are blunted in older adults compared with younger subjects. Finally, the magnitude of the drop in diameter following forearm occlusion correlates with the magnitude of the BAFMD.

## Background

Non-invasive evaluation of brachial artery flow-mediated dilation (BAFMD) has emerged as a useful tool to study vascular function. Celermajer and colleagues [1] are the first to describe this technique, in which high-resolution ultrasonography is used to measure brachial artery diameter at rest and following reactive hyperemia, induced by forearm cuff occlusion. The dilatory response associated with increased flow is thought to be endothelium-dependent, and is subsequently used as a marker of endothelial function. In fact, reduced BAFMD has been found in the presence of numerous CVD risk factors [2-8] and holds predictive value for cardiovascular events [9].

Currently, use of the BAFMD technique is limited mostly to research, but continued refinement of the methodologies may help to enhance its clinical application. For example, seminal work using this technique report BAFMD as absolute and percentage change in vessel diameter from rest to peak dilation, usually 60 seconds following five minutes of forearm occlusion [1,10]. However, advanced technology now makes it possible to collect multiple images of the brachial artery by automatically using the ECG signal as a trigger. Furthermore, computer-based edge detection software allow for semi-automated measurements of the arterial diameter [11]. Accordingly, Bressler and colleagues [12] obtained vessel diameter measurements in an adult sample at 20 second intervals following release of cuff pressure. They found that the average time to reach peak dilatory response was 60 seconds, but values ranged between 40 and 140 seconds. Jarvisalo et al. [13] found similar results in a group of children, indicating that more frequent sampling of the data is needed to identify an individual's true peak response. Assessing variables such as the time taken to reach peak diameter may improve the utility of the BAFMD technique and subsequently allow for better understanding of the physiological processes dictating vascular function.

Hence, the purpose of this study was to examine the temporal pattern of the BAFMD vasoreactivity curve immediately following five minutes of forearm occlusion in a group of younger sedentary men. Also, given that BAFMD declines progressively throughout the lifespan [14] we speculate that age may also influence specific features of the vasoreactivity curve. Therefore a second objective of this study was to compare these features between a sample of young and older men.

## Methods

### Participants

Sixteen healthy, young men, aged 21 to 44 years were recruited to participate in our study. Smokers, those with renal impairment and proteinuria, hepatic impairment, gout, anemia, hypercholestrolemia, hypertension, diabetes, acute medical conditions and active infection were excluded from the study. In addition, brachial artery vascular data from fifteen older men, aged 71 to 100 years, were used to allow for comparison of specific features of the vasoreactivity curves against the younger subjects. The only exclusion criteria for the older adults were individuals in the American Heart Association Class D (i.e., symptoms of cardiovascular and/or metabolic disease at rest). Each participant signed an informed consent approved by the Pennington Biomedical Research Center Institutional Review Board and Louisiana State University.

### Experimental protocol

All brachial artery imaging and analyses were conducted in accordance with the Guidelines set forth by the Brachial Artery Reactivity Task Force [11]. Brachial artery ultrasound measures (Toshiba Power Vision SSA-380A) were obtained with participants in the supine position using a 7.5-MHx linear array transducer prior to, during and following five minutes of forearm occlusion. Prior to scanning, participants were instructed to fast and refrain from exercise and alcohol intake for 12 and 48 hours, respectively. Baseline ultrasound images were obtained after 10 minutes of supine rest. All images were obtained in the longitudinal view, approximately 4 cm proximal to the olecranon process, in the anterior/medial plane. Image depth was initially set at 4 cm gain settings were adjusted to provide an optimal view of the anterior and posterior intimal interfaces of the artery and kept constant throughout. The participant's arm was immobilized and slightly supinated. Forearm occlusion consisted of inflation of a blood pressure cuff, positioned approximately 1 cm distal to the olecranon process, to 240 mm Hg for five minutes. Images for vessel diameter and velocity profiles were obtained at rest, and continuously from the final 30 seconds of occlusion until five minutes following the release of the blood pressure cuff. In addition, blood pressure and heart rate were monitored throughout the imaging process. All ultrasound images were recorded on compact discs for subsequent analysis.

### Data analyses

The Brachial Imager software (Medical Imaging Applications, LLC) was used to analyze the images. Arterial diameters were calculated as the mean distance between the anterior and posterior wall at the blood vessel interface, with the image in diastole, defined as the peak of the R wave on the ECG. Base diameter (BASE) was defined by the average of 30 seconds of data obtained after 10 minutes of resting conditions. Peak dilation (PEAK) was defined (by visual inspection of the arterial diameter curve) as the largest diameter following release of the occluding cuff. Its value was calculated by the average of 10 images (five seconds) surrounding this highest observable peak.

Three other characteristics of the vasoreactivity curve were identified and subsequently analyzed. The pre release diameter (PRE) was defined as the average of 20 seconds of data collected during occlusion, prior to release of the blood pressure cuff. The initial decrease in arterial diameter following cuff release was quantified as the average of 10 images surrounding the smallest diameter (NIL). The magnitude of the initial decrease following occlusion was defined as the absolute (mm) and percent change in vessel diameter from BASE to NIL. Finally, BAFMD was defined as the absolute (mm) and percent change in vessel diameter from BASE to PEAK.

Flow velocity profiles were obtained at rest and immediately following release of the blood pressure cuff. From each velocity profile, the flow velocity integral (FVI)(cm) was manually traced, using Image Pro 4.0 software. The FVI was then divided by the ejection time (s) from that cardiac cycle to subsequently determine the mean velocity (cm·s^-1^). At rest, the average of three velocity profiles was used to calculate resting mean velocity (Vmeanrest). Within 10 seconds of cuff deflation, an average of three velocity profiles was used to calculate mean hyperaemic velocity (Vmeanhyper). The vessel radius (cm) at rest and immediately post occlusion was used in the equation, (Vmean*heart rate)*πr^2 ^to calculate mean blood flows (ml·min^-1^) at rest and hyperemia, respectively. Finally, the mean wall shear rate upon release (Vmeanhyper/Diameter) was calculated according to recently published findings [15].

### Statistical analyses

Independent t-tests were used to compare age groups for descriptive characteristics such as age, weight, height, BMI, resting heart rate, and blood pressure. To examine the main features of the temporal pattern of the brachial artery diameter before and after occlusion, a repeated measure ANOVA (*Feature*: BASE, PRE, NIL, PEAK) was used. Post-hoc comparisons were made using t-tests. To examine the difference in the main features of the temporal pattern between young and old, a 2 (Young and Old) * 4 (BASE, PRE, NIL, PEAK) ANOVA was used. To further appreciate differences between the younger and older subjects a multivariate analysis was performed to examine the magnitude of the decrease in diameter and BAFMD. Finally, associations between features of the temporal pattern of the brachial artery were examined using Pearson Product Moment Correlations.

## Results

### Participant characteristics

None of the participants had any overt signs of disease. Participant characteristics are shown in Table 1. Independent t-tests reveal that the older adults had a higher BMI and systolic blood pressure as compared to their young counterparts (all *p-value*s < 0.05).

**Table 1:** Participant characteristics

	Young	Old	*p *value
	
Variable	Mean ± SD	Mean ± SD	
Age (years)	28 ± 7	85 ± 11	0.001
Weight (kg)	70.0 ± 9.5	71.4 ± 11.0	0.757
Height (cm)	177 ± 7	165 ± 11	0.006
BMI (kg/m^2^)	22 ± 2	26 ± 4	0.003
Resting SBP (mmHg)	114 ± 10	139 ± 4	0.004
Resting DBP (mmHg)	71 ± 8	77 ± 13	0.373
Resting HR (bpm)	68 ± 9	60 ± 6	0.400

Values are means ± SD for 16 young subjects, 15 old subjects BMI, Body Mass Index, SBP, systolic blood pressure, DBP, diastolic blood pressure, HR, heart rate

### Temporal pattern of the vasoreactivity curve

**Figure 1:**
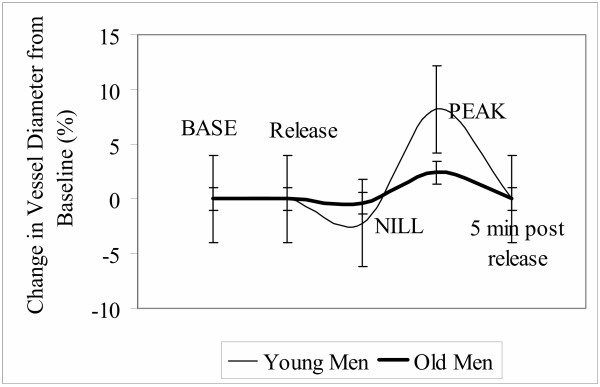
Features of the vasoreactivity curve: Young vs. Old; BASE: base diameter (30 sec average); NIL: lowest diameter post cuff release (5 second average); and PEAK: largest diameter post cuff release (5 second average). Data represents percentage change from baseline.

The temporal pattern and main features of the brachial artery vasoreactivity curve, for the young and old men, are displayed in Figure 1. Following the forearm occlusion a biphasic pattern is evident with an initial significant decrease in the diameter of the vessel (NIL) as compared to the BASE and PRE diameters (*p *= 0.03). No significant difference was observed between BASE and PRE.

After approximately 10 seconds the vessel diameter begins to increase, reaching PEAK at 41 seconds (*p *= 0.001 vs. BASE). Subsequently, the vessel diameter gradually returns toward the baseline value over the next four minutes. Absolute values of BASE, PRE, NIL and PEAK are shown in Table 2. In addition, the magnitude of the decrease and BAFMD are also shown in Table 2.

**Table 2:** Between groups comparison of the main features of the vasoreactivity curve

Features	Young	Old	*p *value
BASE (mm)	3.6 ± 0.5	4.4 ± 0.7	0.002
PRE (mm)	3.5 ± 0.5	4.5 ± 0.6	0.001
NIL (mm)	3.5 ± 0.5	4.4 ± 0.6	0.001
PEAK (mm)	3.9 ± 0.5	4.5 ± 0.7	0.015
Time to NIL (sec)	10 ± 4	14 ± 9	0.126
Time to PEAK (sec)	41 ± 16	59 ± 26	0.297
Magnitude of Decrease (% Δ)	2.2 ± 0.8	0.4 ± 2.1	0.015
BAFMD (% Δ)	7.7 ± 3.5	2.6 ± 1.2	0.001
Resting Blood Flow (ml/min)	131 ± 63	232 ± 131	0.046
Hyperemic Blood Flow (ml/min)	396 ± 95	495 ± 199	0.161
Mean Wall Shear Rate (s^-1^)	191 ± 64	135 ± 63	0.064

Values are mean ± SD

### Comparison of the main features of the temporal pattern between young and old men

Absolute values of the brachial artery diameter at the BASE, PRE, NIL and PEAK time points for the older adults are also shown in Table 2. The 2*4 ANOVA revealed a significant main effect of group such that vessel diameter was greater in the older adults at all points compared to the young (*p *= 0.0001). Moreover, blood flow estimates immediately following cuff release appeared greater in the older subjects (*p *= 0.15), whereas the mean wall shear rates were higher in the Young (*p *= 0.06). Results of the multivariate ANOVA revealed a significant difference between the Young and Old for the magnitude of the decrease in diameter, (2.2% vs. 0.4%, *p *= 0.015) and the BAFMD (7.7% vs. 2.6%, p = 0.001) (see Table 2). The difference in the magnitude of the decrease in diameter was not significant (*p *= 0.14) when the BASE and mean wall shear rate were used as covariates in the model. In contrast, the BAFMD difference remained significant (*p *= 0.004), using the ANCOVA. Finally, no differences were noted for time to reach NIL and PEAK diameters.

### Relation between the magnitude of the NIL and BAFMD

The BASE was inversely associated with BAFMD (r = -0.48, *p *= 0.006). Moreover, the mean wall shear rate was significantly related to BAFMD (r = 0.62, *p *= 0.003) and the magnitude of the decrease in diameter following occlusion (from BASE to NIL) (r = -0.51, *p *= 0.05). Finally, there was also a significant relationship between the magnitude of the decrease and BAFMD (r = -0.44, *p *= 0.04) with approximately 21% of the variability in BAFMD explained by the magnitude of change between BASE and NIL.

## Discussion

This study examined the temporal pattern of brachial artery diameter after five minutes of forearm occlusion. The findings indicate a biphasic pattern whereby immediately following cuff release brachial diameter decreases from baseline. This is followed by a gradual increase in diameter for approximately 41 seconds to a peak and subsequent return toward baseline values. This biphasic pattern is evident in both young and older healthy participants but appears to be significantly blunted in the older group as indicated by differences in the main features of the vasoreactivity curve. Finally, significant relationships were noted between the magnitude of the initial drop and BAFMD, and the shear rate and the magnitude of the initial drop and BAFMD.

### Temporal pattern in young men

Given the hypothesis that endothelial dysfunction is an initiating event in atherosclerosis, a significant amount of research has led to the development of non-invasive tools to assess vasoreactivity in an attempt to identify individuals at risk for vascular disease or to examine the influence of treatment strategies [9]. The underlying assumption of this research is that a sudden increase in shear stress can activate stretch receptors on the endothelial surface, which trigger the release of vasodilatory molecules, and subsequent arterial vasodilation [16].

Probably the most commonly used technique to study this phenomenon is the BAFMD model. Traditionally, BAFMD has been assessed by measuring vessel diameter at baseline and at a specified time point, usually 60 seconds after five minutes of forearm occlusion [11]. The basis for selection of the 60 second time point stems from research that measured the brachial diameter at one-minute intervals following cuff release [11]. The results indicated a peak response at the first minute after release. However, since no measurements were made within the first minute after release, the true peak diameter may not have been observed. Nonetheless, given the evidence that the BAFMD response is related to coronary vasoreactivity [17] and is, generally, reduced in individuals with greater severity of coronary artery stenosis [18], the technique is increasingly viewed as a surrogate marker of cardiovascular health.

Since Coretti's original work, technological advances have allowed more detailed assessment of arterial behavior that question the suitability of a set arterial dilation assessment point. For example, subjects with documented coronary artery disease have been shown to reach peak dilation at approximately 80 seconds [12]. Arterial diameters in this group were sampled every 20 seconds following cuff release and demonstrated a wide individual variation in time to peak (40 to 140 seconds), suggesting this sampling method may be still too large. In fact, we surmised, knowledge of vessel behavior over the entire period of examination may yield more significant information concerning vascular function/health.

The image acquisition used in this study allowed for discovery of a distinct biphasic pattern following the occlusion period. The arterial diameter initially decreases to a nadir at 10 seconds before gradually increasing in size to a peak at 41 seconds post cuff release. To our knowledge, this biphasic pattern has not been previously reported in the literature. The reason for the initial decrease is unclear but must be the consequence of, (1) mechanical, (2) physiological and/or (3) structural mechanisms. Although a definitive answer to this puzzle is beyond the scope of this study we have taken the liberty to speculate on each of these areas. Upon release of the blood pressure cuff there is an immediate and rapid increase in flow velocity of blood through the vessel, which may contribute to a decrease in the vessel diameter due to flow kinetics or the response to a rapidly emptying artery (and concomitant fall in local blood pressure). In fact, a significant association between the Vmean at release and the magnitude of the drop (r = -0.44, *p *= 0.05) in the present study may support this speculation. The immediate decrease could also be the results of a myogenic reflex or constriction to counter the sudden stretch induced by a greater volume of blood perfusing through the vessel. This mechanism is commonly seen in smaller arterioles [19]. However, in the present study there was no association between the estimated hyperaemic blood flow and the magnitude of the drop. Finally, the load bearing properties of the collagen and elastin components of the vascular wall, which influence arterial distensibility, may determine how the vessel responds to sudden changes in intravascular pressure [20]. In this context, a stiffer vessel would experience less recoil in response to a change in pressure compared to a more distensible vessel.

Peak dilation (BAFMD) in our young group was ~8%, which is consistent with the literature in similar populations [1,21,22]. Presumably, the initial delay in vasodilation may be dependent upon changes in blood velocity, endothelial stretch receptor stimulation, intracellular signalling and the release of vasoactive substances [16,23]. Clearly the complex temporal pattern of the post cuff release vasoreactivity curve suggests a significant interplay of many factors that may contribute to the overall vascular response.

### Comparison of the main features of the vasoreactivity curve between young and old

Findings from population-based research indicate BAFMD is clearly influenced by the aging process [4,14]. Celermajer reported a reduction in flow-mediated dilation with increasing age. In men, flow-mediated dilation declined at a rate of 0.21% per year after the age of 40 years. In women, flow-mediated dilation was preserved up till the 5^th ^decade of life, and then declined at a rate of 0.49% per year. Similarly, Herrington et al. [14] report a 0.76% reduction in BAFMD, and approximately a 0.23 mm increase in baseline diameter, for each decade of life after 45 years. The reason for the decline in BAFMD observed may be attributable to reduced nitric oxide-mediated vasodilator response [24] or age related changes in the vascular smooth muscle tone [25]. The structural changes in the vessel may in part explain the progressive increase in baseline diameter seen across the life span. Our results are in agreement with those of Herrington and colleagues. We observed the baseline diameter to be significantly larger (4.4 mm vs. 3.6 mm, *p *= 0.001) and the BAFMD significantly lower in the older adults compared to the younger adults (2.6% vs. 7.7%, *p *= 0.001). Importantly, we also found that the mean wall shear rate following occlusion appeared to be greater in the young (191 ± 64 s^-1^) compared to the old (135 ± 63 s^-1^). Although the observed difference was not statistically significant, the p-value was 0.06. Given our study confirms an inverse relation between vessel size and BAFMD, and a direct association between mean wall shear rate and BAFMD, it raises an important issue; is the change in BAFMD merely a consequence of a change in baseline diameter and/or a change in the signal strength for dilation, rather than a reduction in the mechanisms that contribute to the vasoreactivity response?

In an effort to examine this issue more closely we used an ANCOVA, with the BASE diameter and mean wall shear rate as the covariates. The results revealed that the BAFMD remained significantly lower in the Old (see Figure 1.). These findings suggest the blunted BAFMD in the elderly is, in part, due to a reduced physiological (endothelial) response to the vasodilatory stimulus, and not merely a consequence of a larger resting diameter or smaller hyperemic signal. However, these findings do highlight the importance of considering the role of the baseline diameter on BAFMD, and the need to normalize the reactive hyperemic stimulus for examining BAFMD between groups differing in baseline diameter [26]. Future studies should further examine the mechanisms for the increasing baseline diameter. Some evidence suggests large conduit arteries remodel as a compensation to early atherosclerosis [27]. Other possible mechanisms may include a loss of vascular smooth muscle tone, or a response to an increase in vascular resistance in smaller resistance vessels [28].

In the older group, as in the younger participants continuous monitoring of the vessel diameter throughout the period of reactive hyperemia revealed a biphasic pattern. The reduction in the vessel diameter occurred immediately following the release of the blood pressure cuff, reaching the smallest diameter at approximately 10 seconds, a percentage change from baseline of only 0.4% vs. 2% for the young. The vessel gradually increased in diameter until it reached its peak diameter at approximately 59 seconds (BAFMD = 2%). It is important to note that whereas the BAFMD remained significantly lower in the old, when the BASE diameter and mean wall shear rate were incorporated into the model, the magnitude of the drop was no longer statistically significant (*p *= 0.13) between the groups. Yet, from the findings it is clear that the biphasic pattern is significantly blunted in the old. While it is understood that reduced nitric oxide-mediated vasodilator response is partly responsible for the diminished BAFMD it is less clear why there is a reduction in the magnitude of the drop in vessel diameter following the release of cuff pressure. However, if the decrease in diameter is a consequence of the myogenic reflex or a decrease in distensibility of the vessel, than these mechanisms may also be influenced by the aging process.

### Relationship between magnitude of decrease and BAFMD

Another unique finding of this study was the apparent inverse association between the magnitudes of change from BASE to NIL diameters and BAFMD. Specifically, individuals with the largest drop in diameter immediately following cuff release had the largest BAFMD. We are unsure as to the exact meaning of this association but believe it ought to be considered when examining the overall reactivity of the conduit vessel. For instance, flow mediated dilation has been traditionally thought of as a tool used exclusively to define endothelial function. Accordingly, diminished response to the flow stimulus is typically viewed as dysfunction of the endothelial cells to produce vasoactive substances such as nitric oxide [29]. The decrease in vessel diameter immediately following the release of cuff pressure observed in the present study is probably not endothelial-mediated, as the drop occurs within 10 seconds. Arguably the immediate response is probably faster than the time needed to release an endothelial substance, like endothelin, that could have a vasoconstricting effect in a large conduit artery. Thus, if we assume that the initial decrease is influenced by factors such as the myogenic reflex or the stiffness of the vessel, it is feasible that these same properties also influence the behavior of the vessel during reactive hyperemia. Consideration of these other factors may ultimately enhance the BAFMD technique and subsequently allow for better understanding of the physiological and structural processes dictating vascular function.

## Conclusion

In conclusion, following forearm occlusion, continuous monitoring of the brachial artery reveals a biphasic pattern, marked by an initial decrease and subsequent increase in brachial artery diameter. This biphasic pattern is blunted in older adults and the magnitude of the initial drop in diameter correlates with the magnitude of the vasodilatory response. These unique findings may provide additional information regarding the structural and physiological status of the brachial artery following occlusion and may further enhance the use of the BAFMD model.

## Authors' contributions

DAD: coordinated and executed study design and contributed to the manuscript

AAA: executed study design and contributed to the manuscript

JDA: executed study design, and contributed to the manuscript.

RHW: offered critique of final manuscript

MAW: conceived of study design and contributed to the manuscript

All authors read and approved of the final manuscript

## Competing interests

The author(s) declare they have no competing interest.
